# Microencapsulation of a Commercial Food-Grade Protease by Spray Drying in Cross-Linked Chitosan Particles

**DOI:** 10.3390/foods11142077

**Published:** 2022-07-13

**Authors:** María D. Busto, Yaiza González-Temiño, Silvia M. Albillos, Sonia Ramos-Gómez, María C. Pilar-Izquierdo, David Palacios, Natividad Ortega

**Affiliations:** Department of Biotechnology and Food Science, Area of Biochemistry and Molecular Biology, University of Burgos, Plaza Misael Bañuelos s/n, 09001 Burgos, Spain; dbusto@ubu.es (M.D.B.); ygonzalez@ubu.es (Y.G.-T.); salbillos@ubu.es (S.M.A.); soniarg@ubu.es (S.R.-G.); mcpilar@ubu.es (M.C.P.-I.); dpalacios@ubu.es (D.P.)

**Keywords:** chitosan, Flavourzyme^®^, microencapsulation, protease, spray drying, tripolyphosphate

## Abstract

In this study, the use of spray-drying technology for encapsulating Flavourzyme^®^ (protease–peptidase complex) was evaluated to overcome the limitations (low encapsulation efficiency and no large-scale production) of other encapsulation processes. To the best of our knowledge, spray drying has not been applied previously for the immobilization of this enzyme. Firstly, bovine serum albumin (BSA), as a model protein, was encapsulated by spray drying in chitosan and tripolyphoshate (TPP) cross-linked-chitosan shell matrices. The results showed that the chitosan–TPP microcapsules provided a high encapsulation efficiency and better protein stability compared to the non-crosslinked chitosan microcapsules. The effect of enzyme concentration and drying temperature were tested during the spray drying of Flavourzyme^®^. In this regard, an activity yield of 88.0% and encapsulation efficiency of 78.6% were obtained with a concentration of 0.1% (*v*/*v*) and an inlet temperature of 130 °C. Flavourzyme^®^-loaded chitosan microcapsules were also characterized in terms of their size and morphology using scanning electron microscopy and laser diffractometry.

## 1. Introduction

A traditional strategy with which to accelerate cheese ripening is the use of exogenous enzymes (proteinases, peptidases, and lipases) [1,2]. Nevertheless, the direct addition of proteolytic enzymes to cheese milk causes poor enzyme distribution, whey contamination, lower yield, and poor-quality cheese [3,4,5]. These drawbacks can be overcome by incorporating encapsulated enzymes [4,6].

Flavourzyme^®^ (EC. 3.4.11.1) is a fungal protease-peptidase complex derived from *Aspergillus oryzae* and used for the acceleration of cheese ripening, especially for the debittering and flavour development of cheese [7]. This commercial food-grade protease has already been encapsulated in liposomes [8], gellan, *k*-carrageenan, the high-melting-fat-fraction of milk [4], and nanoliposomes by a heating method [9]. The main limitations of these forms of encapsulation include their low encapsulation efficiency and lack of suitability for large-scale production. Spray drying could be used as an alternative encapsulation technology due to its flexible, continuous, and economical operation [10].

Microencapsulation by means of spray drying is a topic of great interest, as demonstrated by the increasing number of scientific articles published and patent applications submitted in the last decade [11,12,13,14,15,16,17,18]. Spray drying is a preservation and controlled release technology that is commonly applied in the food industry [10,19,20,21], mainly due to being a relatively low-cost technique that is reproducible, rapid, and easy to scale-up, when compared with other microencapsulation technologies [22,23]. Spray drying has been used in the food science field to encapsulate flavours, bioactive and functional compounds, probiotics, enzymes, or even cells [19,20,21,22,23,24,25,26,27,28,29,30,31,32,33]. A crucial step in developing spray-dried microcapsules for food applications is the choice of the encapsulating material, since its properties (i.e., solubility and emulsifying properties) determine the encapsulation efficiency and stability of the microcapsules [34]. One of the most commonly used compounds for the microencapsulation of enzymes is chitosan, thanks to its intrinsic properties, such as biocompatibility and biodegradability and its acceptable level of solubility in water [35,36]. Chitosan has been used to encapsulate enzymes such as α-amylase [37], invertase [37], lipase [38], β-galactosidase [39], glucose oxidase [40], and inulinase [41]. The different chitosan-based encapsulation techniques applied in enzyme immobilization (layer-by-layer self-assembly, emulsification, hydrogels, film, ionotropic gelation, and spray drying) have recently been reviewed by Zhang et al. [42]. The ionic gelation technique (reversible physical crosslinking via electrostatic interactions between the anionic groups and polycationic amino groups of chitosan) has been widely used for the nanoencapsulation of food components by chitosan [43]. A bio-compatible cross-linker for chitosan is tripolyphosphate (TPP) anions [44]. Previous works have shown that enzymes can successfully be microencapsulated using TPP-cross-linked chitosan microparticles by means of spray drying [45,46].

However, to the best of our knowledge, nothing has been published to date regarding the encapsulation of Flavourzyme^®^ using the spray-drying methodology. Thus, the objective of this study was to investigate, for the first time, the microencapsulation of this commercial food-grade enzyme by means of spray drying. Preliminary studies were also conducted with bovine serum albumin (BSA), as a model protein, in order to compare crosslinked and non-crosslinked encapsulation, and for the control of protein release from the capsules. Additionally, the influence of Flavourzyme^®^ concentration and drying temperature on the activity of the encapsulated enzyme and the size and morphology of the resulting microparticles was also investigated.

## 2. Materials and Methods

### 2.1. Materials

Chitosan (high molecular weight, ≥75% deacetylated) was purchased from Fluka Chemie (Buchs, Switzerland), Flavourzyme^®^ was purchased from Novozymes (Bagsværd, Copenhagen, Denmark), and sodium tripolyphosphate (TPP) and bovine serum albumin (BSA) were obtained from Sigma-Aldrich Co. (St. Louis, MO, USA). All other chemicals were of analytical grade. The water used was purified using a Milli Q water purification unit (Millipore Corp., Bedford, MA, USA).

### 2.2. Preparation of Chitosan Microparticles by Spray Drying

#### 2.2.1. Non-Cross-Linked Chitosan Microcapsules

Chitosan (0.5%, *w*/*v*) was dissolved in 250 mL of an aqueous solution of acetic acid (2%, *v*/*v*). After the complete dissolution of the polymer, 250 mg of BSA was added. This solution was spray dried by a Mini Spray Dryer Buchi B-290 (Büchi Labortechnik AG, Flawil, St. Gallen, Switzerland), which is a laboratory spray-drier equipped with a 2-fluid nozzle with a 1.5 mm nozzle cap and a cyclone in an open-mode configuration. The operation conditions were as follows: an aspirator rate (drying air flowrate) of 90%, liquid feed flowrate of 6.4 mL/min, atomizing gas flowrate (N_2_) of 414 NL/h, inlet temperature fixed at 130 °C, and outlet temperature fixed at 66 ± 1 °C.

#### 2.2.2. Cross-Linked Chitosan Microcapsules

TPP has been described as a useful cross-linker for chitosan cross-linking [47]. An ex situ cross-linking method was applied whereby chitosan and TPP were pre-mixed before being spray dried with the protein/enzymatic solution. There is an upper limit on the chitosan concentration that can be practically used during ex situ cross-linking. Kašpar et al. [48] described that for chitosan concentrations above approximately 0.5% (*w*/*w*), a TPP droplet immediately changes into an insoluble coagulate upon contact with the chitosan solution, which makes it difficult to spray due to nozzle clogging. Therefore, in this procedure, 10 mL of a TPP solution (1%, *w*/*v*) was dropped with a syringe into a chitosan solution (0.5%, *w*/*v*) under magnetic agitation to obtain a TPP/chitosan ratio of 0.08. Afterwards, BSA (250 mg) or Flavourzyme^®^ (100, 250, 400 µL) were added to the colloidal suspension of the cross-linked chitosan–TPP mixture and spray-dried by a 2-fluid nozzle in order to form the microcapsules.

For BSA encapsulates, the conditions were: aspirator rate, 90%; liquid feed flowrate, 6.4 mL/min; atomizing gas flowrate (N_2_), 279 NL/h; inlet temperature, 130 °C; and outlet temperature, 64 ± 1 °C. For the Flavourzyme^®^ microcapsules, the tested operating conditions are shown in Table 1.

### 2.3. Particle Size and Morphology Characterization

The particle size and size distribution were both measured by laser diffractometry in a Mastersizer 2000 (software version 5.60) (Malvern Instruments, Ltd., Malvern, Worcestershire, UK). The particle size was expressed as the volume median diameter (dv50, which represents the maximum particle diameter below which 50% of the sample volume exists), which is one of the most commonly reported particle size parameters in spray-dried powders. Microcapsules (50 mg) were pretreated by dispersion in 50 mL of distilled water and placed in an ultrasound water bath for 5 min in order to break apart eventual temporary agglomerates. The measurement was repeated 3 times for every sample.

The shape and surface morphology of the obtained microparticles were examined by a scanning electron microscope (SEM) JEOL JSM-6400 LV (JEOL Ld., Akishima, Tokyo, Japan) coupled to an X-Max energy-dispersive X-ray spectroscopy system. Samples were coated by a thin layer of gold before the analysis. SEM micrographs were obtained at an acceleration voltage of 20 kV and a magnification of 10,000×. The particle analyser plugin of ImageJ was used to retrieve the particle diameters from the SEM images.

### 2.4. Evaluation of Enzymatic Activity

The protease activity of Flavourzyme^®^ was determined using the method described by Ortega et al. [49]. In brief, a reaction mixture of 3 mL of 1% (*w*/*v*) of casein dissolved in 0.1 M tris-borate buffer (pH 8.1) and 0.6 mL of enzyme solution (free or encapsulated), was incubated at 50 °C with constant shaking at 150 rpm for 10 min. The reaction was stopped with ice and then 1.2 mL of trichloroacetic acid (TCA) was added to precipitate the non-hydrolysed protein. An aliquot of 1 mL was added to 3 mL of alkaline reagent, which contains 1 mL of sodium potassium tartrate (1%, *w*/*v*), 1 mL of cupric sulfate (0.5%, *w*/*v*), and 50 mL of sodium carbonate (2%, *w*/*v*) in NaOH 0.1 M. After 15 min, 1 mL of Folin–Ciocalteau reagent (33%, *v*/*v*) was added. The assay mixture was allowed to stand for 45 min at room temperature (22 to 25 °C) and the absorbance was measured at 700 nm in a spectrophotometer. Controls in which the enzyme solution was added after precipitation with TCA were assayed in all cases to deduct any non-enzymatic activity. A standard curve of tyrosine in the range of 0–500 μM was plotted. All the enzymatic assays were performed in triplicate.

One unit of protease activity (U) was defined as the amount of enzyme required to release a number of TCA-soluble products, which have the same colour with the phenol reagent as 1 μmol of tyrosine.

The activity yield (*AY*) in the Flavourzyme^®^ encapsulation was calculated as described in Equation (1).

(1) $$AY\;\left(\%\right)=\frac{Specific\;activity\;of\;the\;microcapsules\;\left(U/mg\;protein\right)}{Specific\;activity\;of\;the\;feed\;sample\;\left(U/mg\;protein\right)}\times 100$$

### 2.5. Evaluation of Protein Encapsulation and Release

Protein-loaded chitosan microparticles (10 mg of BSA-loaded microcapsules or 50 mg of enzyme-loaded microcapsules) in 30 mL of 0.01 M phosphate-buffered saline (PBS, containing 0.01 M Na_2_HPO_4_, 0.002 M KH_2_PO_4_, 0.137 M NaCl, and 0.0027 M KCl) solution at pH 7.4 were separated from the solution by stirring at 1000 rpm at room temperature for 5 min. The protein content in the supernatant was measured at 750 nm using the Lowry quantitative protein assay, as recommended in the original method for an expected range of concentration above 25 μg/mL [50]. The encapsulation efficiency (*EE*) in the BSA or Flavourzyme^®^ preparations was calculated in triplicate using Equation (2).

(2) $$EE\;\left(\%\right)=\frac{Protein\;released\;\left(mg\right)}{Protein\;in\;the\;feed\;\left(mg\right)\times Dry\;extract\;yield}\times 100$$

Protein release from the microcapsules was determined by the method suggested by Gan and Wang [51]. The samples (10 mg or 50 mg of BSA- or enzyme-loaded microcapsules, respectively) were transferred to a flask with 30 mL of PBS. The suspension was placed in a water bath maintaining the temperature constant at 37 ± 1 °C. At specified collection times (0, 1, 3, 5, 7, and 24 h), 1 mL of sample was removed from the flask for the protein assay or 0.6 mL for enzymatic activity measurement. The flask was replenished with the same volume of fresh PBS. The released protein mass *M_i_* at time *i* was calculated from Equation (3).

(3) $$M_{i}=C_{i}V+\sum^{\;}C_{i-1}\times V_{s}$$

where *C_i_* is the concentration of protein in the release solution at time *i*, *V* is the total volume of release solution, and vs. is the sample volume.

Dry extract yield was calculated as the percentage ratio of the weight of powder collected in the sample flask, after spray drying, to the corresponding weight of solids in the feed sample [52].

### 2.6. Differential Scanning Calorimetry (DSC)

DSC experiments were carried out on a Nano DSC Series III system (TA Instruments Inc., Eschborn, Germany) with a temperature scan rate of 1 °C/min from 20 °C to 100 °C. Before scanning, the solutions were degasified for 30 min with a TA degassing station and the samples filtered through nylon filters of 0.45 μm pore size. The thermograms recorded were analysed with the NanoAnalyze 2.0 software. The buffer used for this assay was PBS at pH 7.4. The buffer-buffer baseline was run at least five heating–cooling cycles until the heating was reproducible and then subtracted from the sample data. DSC was applied to the BSA liberated from the microcapsules and compared to a BSA standard. The thermodynamic parameters were calculated according to the molar protein concentration in each sample and expressed in international standard units. The Flavourzyme^®^ commercial preparation contains a complex enzymatic mixture and, therefore, was not suitable for obtaining calorimetric parameters.

### 2.7. Statistic

Analysis of variance was applied to data using Statgraphics Centurion 18. Data in the tables and figures are given as mean values with the standard errors of the mean. All determinations were replicated three times. To compare significant differences in means, the Duncan’s test, at *p* < 0.05, was used, unless otherwise stated.

## 3. Results and Discussion

Traditional cheese ripening involves a series of complex microbiological and biochemical process that occur at a very slow rate. One of the most specific methods reported to accelerate cheese maturation is the addition of encapsulated proteases (such as Flavourzyme^®^) [36]. The enzyme containing in the microcapsules is physically separated from the milk casein during cheese making and released during ripening when the capsule breaks down [4]. In this paper, the encapsulation of Flavourzyme^®^ by spray drying is investigated as an alternative technique to solve the main limitations (low encapsulation efficiency and no large-scale production) previously described for the encapsulation of this enzyme [4,8,9].

### 3.1. BSA-Loaded Chitosan Microparticles Prepared by Spray Drying

#### 3.1.1. BSA Encapsulation, Protein Release and Stability

The protein was encapsulated in the chitosan microparticles, as explained in the Material and Methods section, with an encapsulation efficiency of 81.8 ± 1.7% and 80.8 ± 5.9% for non-crosslinked and crosslinked microcapsules (*p* = 0.163), respectively. In this regard, Kusonwiriyawong et al. [53] described an entrapment efficiency of BSA-loaded non-crosslinked chitosan microparticles by spray drying ranging between 50.5 and 86.7%, lower than the values obtained in this experiment, using a chitosan solution of 1%, a 120 °C inlet temperature, and a previous model of the Büchi spray dryer. On the other hand, the EE of this protein-loaded in alginate microspheres, prepared by spraying, was lower than 66.5% [35]. Gan and Wang [51] obtained an EE of 88.3% using TPP as a crosslinking agent to form chitosan–BSA–TPP nanoparticles by coacervation.

**Figure 1:**
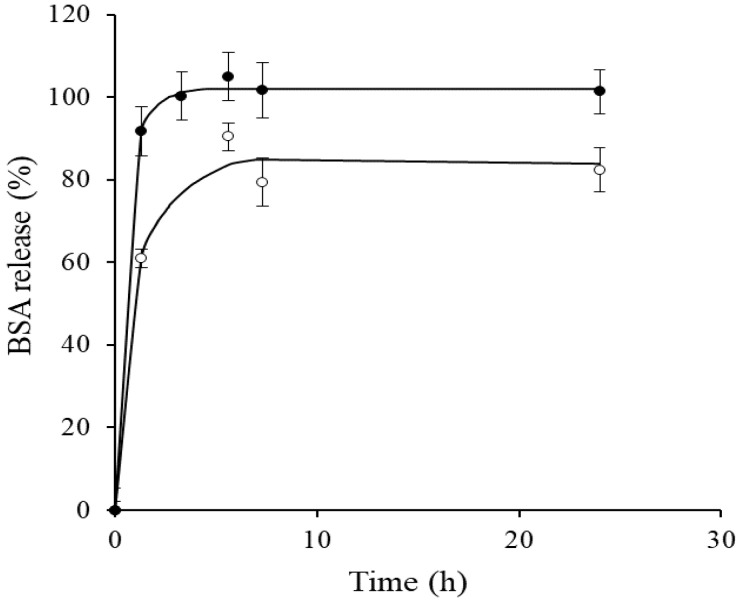
Bovine serum albumin (BSA) release profile from chitosan microcapsules (○) and chitosan−TPP-microcapsules (•). Release medium: Phosphate-buffered saline (PBS) at pH 7.4 and 37 ± 1 °C.

Protein release studies from both types of microcapsules (chitosan and chitosan–TPP) in PBS are shown in Figure 1. Three hours were enough in order to liberate all the protein from both kind of microencapsulates. It seemed that the use of a cross-linking agent did not produce any stabilizing effect on the microcapsules and that the release profile was similar or even faster for the cross-linked encapsulates. Therefore, three hours were chosen as a suitable release protein time for consequent experiments. Different studies have demonstrated that the chitosan molecular weight, the concentration of chitosan, as well as the chitosan to TPP mass ratio are major factors in determining protein release from chitosan-based microparticle systems [51,54]. Nevertheless, the detailed controlling parameters and specific conditions ruling this process are still unknown and often contradictory in the various studies published. Protein release in this type of system is characterized by an initial rapid release (burst effect) within the first 3–6 h [51]. Jiang et al. [27] found that α-amylase encapsulated in Ca-alginate coacervate microparticles and in chitosan–calcium–alginate complex coacervate matrices reached the maximum release value at 3 and 5 h, respectively. For spray-dried BSA chitosan particles, which present some surface activity, it is expected that the protein would be preferably concentrated on the droplet surface, and assuming that the diffusion will be slower than the drying speed, this would explain the fast release observed experimentally from microencapsulates [55].

**Figure 2:**
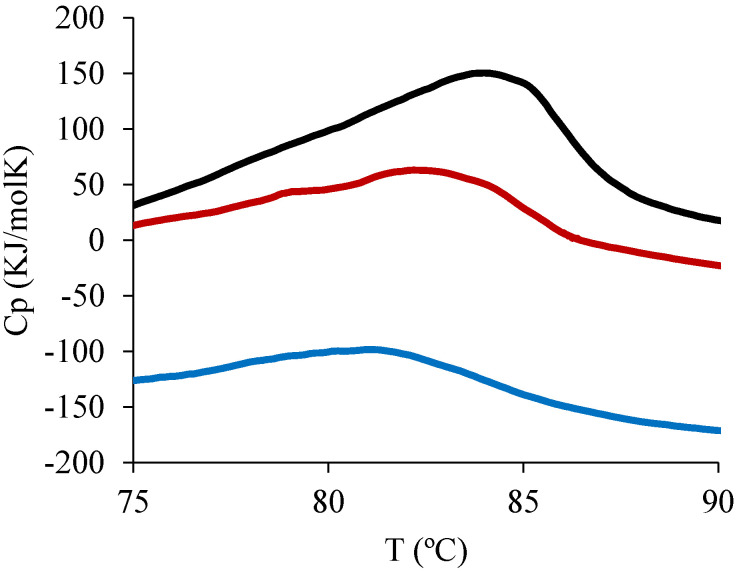
Thermograms of bovine serum albumin (BSA) samples representing the heat capacity (cp) versus heating temperature (°C) of native BSA (black line), BSA released from chitosan non-crosslinked microparticles (red line) and BSA released from chitosan−TPP crosslinked microparticles (blue line). TPP: tripolyphoshate.

The influence of spray drying on the stability of the encapsulated protein was evaluated using DSC. For this purpose, samples of native BSA and BSA released from both types of microcapsules were studied. This methodology is widely used for the study of thermal protein denaturation [56]. The protein denaturation temperature (Td) is defined as the temperature coinciding with the peak maximum in the endotherm, while the enthalpy of denaturation (∆H) can be estimated from the area under the peak in the endotherm [57]. Both thermodynamic parameters are an indication of the protein stability. The thermograms obtained can be seen in Figure 2.

The experimental Td and ∆H values obtained for native BSA and BSA released from chitosan microcapsules and chitosan–TPP microcapsules were 84.0 °C, 81.3 °C, and 82.2 °C and 992 kJ/mol, 282 kJ/mol, and 550 kJ/mol, respectively. The shift towards lower Td and ∆H values for encapsulated protein samples suggests that the atomization process decreases the protein stability, producing a loss of native protein conformation and therefore reducing the energy required for protein denaturation in the DSC experiment. This effect seemed to be smaller for BSA-loaded crosslinked-particles (chitosan–TPP particles) compared to their non-crosslinked counterparts, with this sample having higher Td and ∆H values closer to the values obtained for the native BSA protein. The native BSA ∆H value obtained in the present work (in 0.01 M PBS, pH 7.4) was similar to the one described by Yamasaki and Yano [57] for BSA in 0.1 M NaCl at pH 7.0, with the difference being explained by the non-identical analytical conditions, as the BSA concentration and the ionic strength used for the analysis are significant factors that strongly affect the protein denaturation process.

#### 3.1.2. BSA–Chitosan Particles Morphology and Size

**Figure 3:**
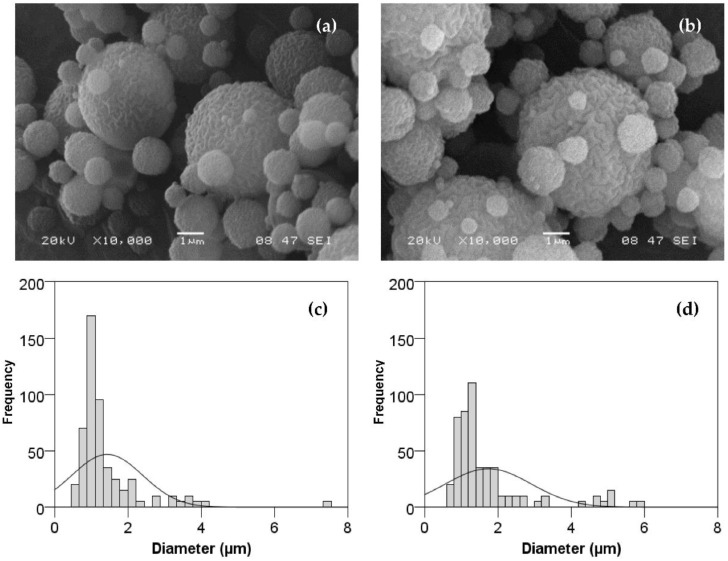
SEM images and size distribution (determined by ImageJ) of BSA loaded in chitosan microcapsules (**a**,**c**) and chitosan−TPP microcapsules (**b**,**d**).

The size and shape of the microencapsulates were studied using scanning electron microscopy and laser diffractometry. The morphological characteristics of the microparticles obtained by SEM and the size distribution determined by ImageJ are shown in Figure 3. Both types of microcapsules had a spherical shape with a rough surface and presented low levels of aggregation. Nevertheless, the microcapsules of chitosan crosslinked with TPP showed more intricate and deeper grooves. At this point, there is discrepancy among authors, with Desai and Park [58] observing a greater roughness on the surface of the microcapsules when the concentration of cross-linking agents increased (from 1 to 2%); on the contrary, Kašpar et al. [48] found that particles of chitosan cross-linked with TPP (TPP/chitosan ratio of 0.09) presented a smoother surface.

Particle size analysis revealed that chitosan microcapsules showed a median size (dv50) of 0.26 ± 0.01 µm, whereas the cross-linking agent produced approximately doubled-sized microcapsules with a dv50 of 0.50 ± 0.02 µm (*p* < 0.000). The chitosan−TPP ratio affects not only the particle morphology but also the particle size distribution. In fact, Kašpar et al. [48] previously reported that a higher level of TPP/chitosan at a constant chitosan concentration will generally yield a wider particle size distribution and a higher mean size. Moreover, a cross-linking ratio of 0.09 for a 2-fluid nozzle (ex situ cross-linking) will lead to a less regular shape—the so-called collapsed balls [48]. Among others, the parameters that affect morphology and particle size in spray drying are the coating material, the diffusion and surface activity of the dissolved material, the solid content in the feed, the droplet size, and the drying conditions [59], as well as the pump rate [60]. For example, BSA loaded in alginate microspheres prepared by spraying showed sizes between 59.2 and 90.2 μm [61]. In another study conducted under different experimental conditions (1% chitosan, inlet temperature of 120 °C, outlet temperature 50–53 °C, and undisclosed flowrate), the size of microparticles of chitosan with BSA used as model antigens obtained by spray drying was about 3 μm [55].

After these initial experiments with a model protein such as BSA and based on the DSC results, which suggested an apparent greater protection conferred to the protein during the spray-drying process by the chitosan cross-linked with TPP encapsulates, this type of coating material was chosen for subsequent Flavourzyme^®^ encapsulation experiments.

### 3.2. Flavourzyme^®^-Loaded Crosslinked Chitosan Microparticles Prepared by Spray Drying

#### 3.2.1. Effect of Enzyme Concentration and Inlet Temperature on Encapsulation Efficiency

In the first experiment, at a 130 °C inlet temperature, the encapsulation efficiency was evaluated by varying the concentration of enzymatic preparation at three different levels: 0.04, 0.10, and 0.16% (*v*/*v*) (Table 2). The encapsulation efficiency achieved for the enzyme-loaded chitosan–TPP microparticles ranged between 62 and 79%, although no significant differences between the EE were observed.

**Figure 4:**
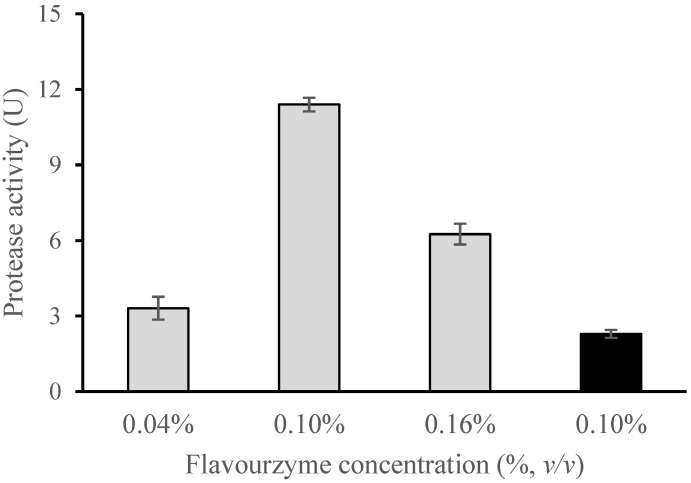
Effect of enzyme concentration on the protease activity of the spray-dried chitosan Flavourzyme^®^ microparticles. Gray and black columns indicate the inlet temperatures of 130 °C and 160 °C, respectively. Errors bars represent the standard deviation.

A parameter of special interest is the activity yield. The mechanical and thermal stress experienced by the proteins during the spray-drying process may cause a significant loss of catalytic activity caused by changes in the secondary and tertiary structures of these macromolecules [52]. In this regard, the best result in our study for Flavourzyme^®^ was obtained with a concentration of enzyme of 0.1% (*v*/*v*), reaching an activity yield of 88.0% (11.4 U), while for concentrations of 0.04% and 0.16% this parameter was reduced to 25.6% (3.3 U) and 60.8% (6.3 U), respectively (Table 2 and Figure 4). Spray drying has been used for the encapsulation of other enzymes, such as alcohol dehydrogenase, with an activity yield of 66% obtained using mannitol as a coating material [62], peptidases from *Eupenicillium javanicum* in maltodextrin with an activity yield of 66.12% [52], or laccase which was encapsulated into a cross-linked chitosan matrix with a retention of about 80% of its enzymatic activity [48].

Flavourzyme^®^ has been encapsulated before in liposomes with very poor activity yields of between 21.9 and 26.5% [9,63], and in *k*-carrageenan, gellan, high-melting-fat-fraction, or glyoxyl agarose beads, with results ranging between 38.9 and 51% [4,64]. Anjani et al. [3] reported an activity yield of 70% when Flavourzyme^®^ was encapsulated in selected alginate or poly L-lysine polymers and gelled with CaCl_2_ containing 0.1% (*w*/*v*) chitosan.

Since the enzyme concentration of 0.1% (*v*/*v*) presented the best results relating to activity yield, this concentration was chosen for the further evaluation of the effect of the inlet temperature and its relation to the microencapsulation efficacy. Two input temperatures commonly used in spray-drying systems, 130 °C and 160 °C, were applied (Table 2). The selection of only two temperatures is justified on the grounds that temperatures lower than 130 °C lead to a sticky product that makes the formation of microencapsulates technologically complicated and temperatures above 160 °C may produce strong denaturing effects on enzymes [48]. By comparing the values of the encapsulation efficiency of the enzyme and activity yield, we observed that there was an important reduction in both parameters when the inlet temperature increased from 130 °C to 160 °C (Table 2). This result is in line with those reported by Shiga et al. [62] and Kăspar et al. [48]. The inlet temperature will affect the outlet air temperature from the spray dryer extraction cyclone. If the inlet temperature is set to 160 °C, the outlet temperature will be raised to 85 °C and quite a high level of enzymatic denaturation can be expected according to the partial characterization conducted by Merz et al. [65] of up to eight of the enzymes included in the commercial preparation Flavourzyme^®^. This will be accompanied by a decrease in the enzymatic activity, consistent with the much lower activity yield obtained.

#### 3.2.2. Effect of Flavourzyme^®^ Concentration and Inlet Temperature on Microparticle Size and Morphology

**Figure 5:**
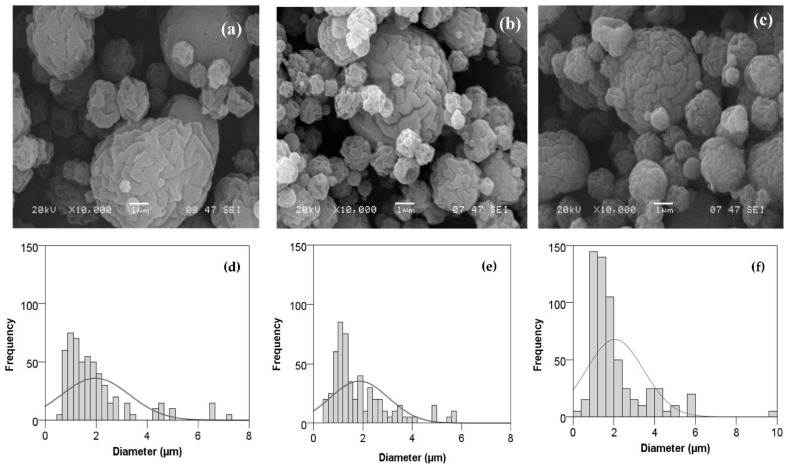
SEM images and size distribution (determined by ImageJ) of Flavourzyme^®^ loaded in chitosan−TPP microcapsules at 130 °C inlet temperature. Enzyme concentration in % (*v*/*v*): 0.04 (**a**,**d**), 0.1 (**b**,**e**), and 0.16 (**c**,**f**).

The SEM images of the Flavourzyme^®^ microcapsules obtained at different enzyme concentrations are presented in Figure 5. All particles had a similar morphology but with some differences. Thus, the encapsulation of enzyme yielded spherical microparticles with some distortions. The surface was rougher and showed more intricate grooves when higher concentrations of enzyme (0.16%) (Figure 5a) were entrapped compared to 0.1% and 0.04% of enzyme (Figure 5b,c, respectively). Furthermore, as the enzyme concentration increased, a smoother and more uniform wall of the microparticles was observed. This may be due to the presence of sucrose in the formulation of the commercial enzyme. The sucrose contained some water molecules linked to its own structure, filling the internal empty space of the microparticles, preserving the hydration, and avoiding depressions on the surface [66].

**Figure 6:**
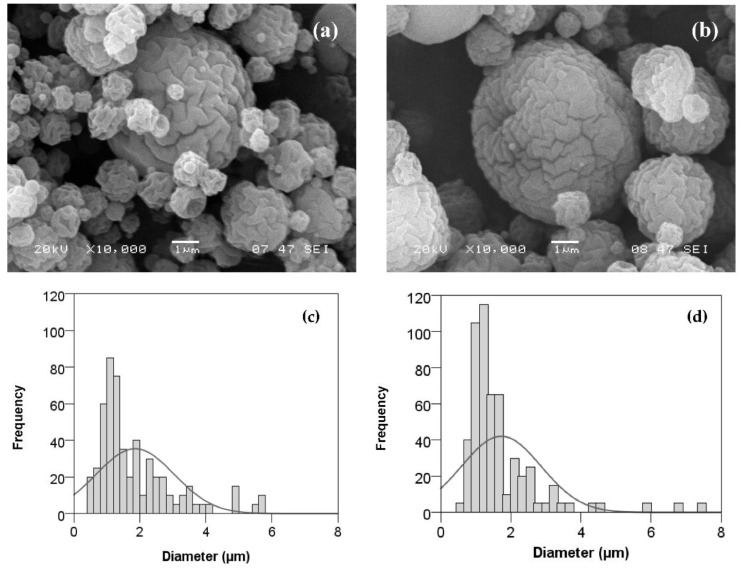
SEM images and size distribution (determined by ImageJ) of 0.10% (*v*/*v*) Flavourzyme^®^ loaded in chitosan−TPP microcapsules at 0.5% (*w*/*v*). Inlet temperature: 130 °C (**a**,**c**) and 160 °C (**b**,**d**).

Outlet air temperature is one of the most critical parameters that affect the product morphology, affecting aspects such as surface roughness, density, and particle size [16]. When the inlet temperature increased from 130 °C to 160 °C, the outlet temperature was also increased from 62 °C to 83 °C (Table 1). As shown in Figure 6, the particles were more regular in shape, with grooves being less defined and sometimes showing the formation of surface collapse at the highest temperature. Likewise, Maas et al. [67] have also found that spray-dried powders at lower outlet temperatures produced higher percentages of more spherical particles without sunken areas on the surface. On the other hand, the median diameter dv50 of the particles measured by the Mastersizer was 503.78 ± 0.02 μm at 130 °C and 387.5 ± 93.32 μm at 160 °C (*p* = 0.106), including particle aggregates, as will be explained later. Other authors have also reported a reduction in particle size with increasing temperature in the spray-drying process [68,69].

**Figure 7:**
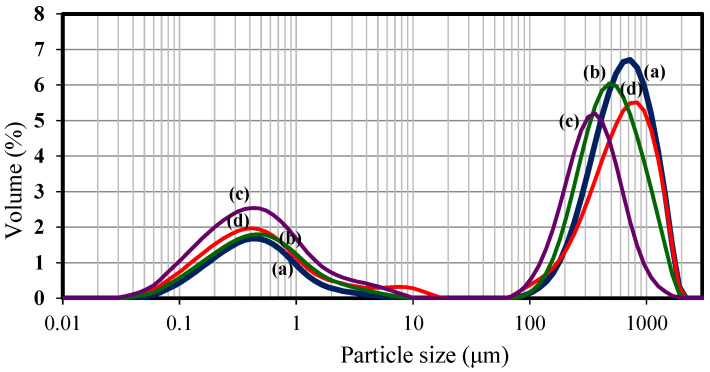
Influence of enzyme concentration and inlet temperature (T inlet) on the particle size distribution of Flavourzyme^®^ chitosan−TPP microcapsules: (a) 0.04% (*v*/*v*), T_inlet_ = 130 °C; (b) 0.10% (*v*/*v*), T_inlet_ = 130 °C; (c) 0.16% (*v*/*v*), T_inlet_ = 130 °C; (d) 0.10% (*v*/*v*), T_inlet_ = 160 °C.

The particle size determined by laser diffractometry showed a clear bimodal distribution, with a group of small particles between 0.1 and 1.1 μm, with the distribution centred around 0.4 μm, and a second group between 100 and 1000 μm (Figure 7). Bimodal distributions were also observed by Katsarov et al. [60] for spray-dried microspheres formed by non-cross-linked chitosan. This bimodal distribution indicated the presence of single particles and agglomerates that have not been dispersed completely [55], which was also confirmed by SEM images, where small and big particles could be observed to have formed clusters due to the cohesion forces keeping them together. The maximum of each peak in the group of particles between 100 and 1000 μm shifted slightly from a higher to smaller particle size, as the Flavourzyme^®^ percentage was increased from 0.04% to 0.10% and 0.16%, keeping the inlet temperature constant at 130 °C and the level of chitosan at 0.5% (*w*/*v*). This indicated that, at 0.16%, there was less formation of aggregates, and the size of these conglomerates was much lower compared to that of the particles formed at a level of 0.04% of enzymatic complex (Figure 6). Only for the sample at 0.10% and the inlet temperature of 160 °C did a third small distribution around 10 μm seem to appear, although this was very small in percentage, while the second distribution was centred at the highest particle size of 900 μm.

The morphology and particle size during the spray-drying process depend on several parameters, such as the droplet size, which depends on the nozzle diameter, the drying conditions in the cyclone, and the solid content in the feeding solution, as well as on the diffusion and surface activity of the dissolved material [59]. If the drying process occurs too fast, there is no time for the dissolved components to diffuse to the centre of the droplet and a shell will be formed, generating more or less hollow particles, which can also lead to an exit hole for gas being entrapped, with a wrinkled morphology similar to that of a deflated ball [55].

## 4. Conclusions

This study aimed at preparing Flavourzyme^®^-loaded chitosan microparticles using spray-drying technology. Thus far, spray drying has not been reported as an encapsulation method applied to Flavourzyme^®^. The results revealed that spray drying by a standard two-fluid kinetic nozzle, using TPP a cross-linking agent, provided a high encapsulation efficiency and a better protein stability. The results also showed important differences in the encapsulation efficiency and activity yield depending on the enzyme concentration and inlet temperature. The maximum protease activity in the microparticles was obtained with 0.10% (*v*/*v*) Flavourzyme^®^ at 130 °C.

In light of our findings, it can be concluded that Flavourzyme^®^ was successfully encapsulated into the cross-linked chitosan matrix with a high retention of protease activity when a suitable enzyme concentration and temperature were applied. Considering that spray drying is a scalable process that can be operated continuously if required, the loading of Flavourzyme^®^ in chitosan–TPP microparticles could be an interesting option for the application of this commercial protease in food processing—e.g., the acceleration of cheese ripening.

## Figures and Tables

**Table 1:** Operating conditions in Flavourzyme^®^ encapsulation with Mini Spray Dryer Buchi B-290 (aspirator rate 90%, liquid feed flowrate 6.4 mL/min).

Flavourzyme^®^ Concentration (%, *v*/*v*)	Inlet Temperature (°C)	Outlet Temperature (°C)	N_2_ Flowrate (NL/h)
0.04	130	57 ± 1	334.6
0.10	130	62 ± 1	334.6
0.16	130	54 ± 1	334.6
0.10	160	83 ± 1	345.8

**Table 2:** Effect of enzyme concentration and inlet temperature on the retention of protease activity in spray-dried chitosan–TPP microparticles ^1^.

Flavourzyme^®^ Concentration (%, *v*/*v*)	Inlet Temperature (°C)	Encapsulation Efficiency (*EE*, %)	Activity Yield ^2^ (*AY*, %)
0.04	130	78.5 ± 11.0 ^β^	25.6 ± 3.5 ^α^
0.10	130	78.6 ± 1.7 ^β^	88.0 ± 2.1 ^δ^
0.16	130	62.4 ± 2.1 ^β^	60.8 ± 4.6 ^γ^
0.10	160	40.6 ± 5.1 ^α^	34.2 ± 0.4 ^β^

^1^ Operating conditions indicated in Table 1. ^2^ Specific activity-feed sample = 0.382 U/mg. Different Greek letters within the same column indicate significant difference (*p* < 0.05).

## Data Availability

The data presented in this study are available on request from the corresponding author.
